# The Immune and Inflammatory Basis of Acquired Pediatric Cardiac Disease

**DOI:** 10.3389/fcvm.2021.701224

**Published:** 2021-07-27

**Authors:** Elysa Jui, Kavya L. Singampalli, Kevin Shani, Yao Ning, Jennifer P. Connell, Ravi K. Birla, Paul L. Bollyky, Christopher A. Caldarone, Sundeep G. Keswani, K. Jane Grande-Allen

**Affiliations:** ^1^Department of Bioengineering, Rice University, Houston, TX, United States; ^2^Medical Scientist Training Program, Baylor College of Medicine, Houston, TX, United States; ^3^Laboratory for Regenerative Tissue Repair, Division of Pediatric Surgery, Department of Surgery, Baylor College of Medicine and Texas Children's Hospital, Houston, TX, United States; ^4^John A. Paulson School of Engineering and Applied Sciences, Harvard University, Cambridge, MA, United States; ^5^Division of Infectious Diseases, Department of Medicine, Stanford University School of Medicine, Stanford, CA, United States; ^6^Division of Congenital Heart Surgery, Departments of Surgery and Pediatrics, Baylor College of Medicine and Texas Children's Hospital, Houston, TX, United States

**Keywords:** pediatric heart disease, pediatric vascular disease, immune response, inflammation, cardiac remodeling

## Abstract

Children with acquired heart disease face significant health challenges, including a lifetime of strict medical management, multiple cardiac surgeries, and a high mortality risk. Though the presentation of these conditions is diverse, a unifying factor is the role of immune and inflammatory responses in their development and/or progression. For example, infectious agents have been linked to pediatric cardiovascular disease, leading to a large health burden that disproportionately affects low-income areas. Other implicated mechanisms include antibody targeting of cardiac proteins, infection of cardiac cells, and inflammation-mediated damage to cardiac structures. These changes can alter blood flow patterns, change extracellular matrix composition, and induce cardiac remodeling. Therefore, understanding the relationship between the immune system and cardiovascular disease can inform targeted diagnostic and treatment approaches. In this review, we discuss the current understanding of pediatric immune-associated cardiac diseases, challenges in the field, and areas of research with potential for clinical benefit.

## Introduction

Cardiovascular diseases (CVDs) comprise a group of disorders that affect the structure and/or function of the heart, including coronary artery disease, arrhythmia, peripheral arterial disease, and congenital heart disease (CHD), among others. Statistics indicate that a large portion of the health burden of CVD is concentrated in adults. However, there is also a significant burden of both acquired and congenital heart disease in the pediatric population, each accounting for hundreds of thousands of childhood deaths annually (1). CHD typically refers to structural disease that forms *in utero*, whereas acquired cardiovascular disease develops after birth. The list of acquired pediatric cardiovascular diseases is extensive, including rheumatic heart disease, myocarditis, vasculopathies, and cardiomyopathy (2); these conditions are often medically managed with serial imaging and medications (3).

Both congenital and acquired heart disease impose significant morbidity on children, including ICU stays, complex medical management, and a high risk of mortality (4). These diseases also impose long-term ramifications, including a risk of myocardial ischemia, valve damage, arrhythmia, and cardiomyopathy (2, 5). Furthermore, with the recent outbreak of COVID-19, it has been shown that pre-existing cardiovascular disease significantly increases the risk of mortality in COVID-19 patients (6), underscoring the importance of addressing these conditions.

Investigating the role of the immune response in the pathogenesis of CVD may be beneficial in children, as it has proven to be a fruitful avenue in adult cardiac disease. In adults, the role of the immune system has been extensively studied in valve disease and post-ischemic remodeling (7, 8). For example, after a myocardial infarction (MI), the inflammatory and cellular immune responses play a role in replacing damaged cardiomyocytes with scar tissue (9). Additionally, some cardiac arrhythmias following an MI have been associated with the systemic presence of inflammation and an increase in the presence of cardiac macrophages, supporting the involvement of macrophage-fibroblast crosstalk in the development of arrhythmias (10). Similar mechanisms may be relevant to pediatric cardiovascular disease as well, since changes such as remodeling, conduction disorders, and fibrosis are not unique to adults (9).

**Figure 1:**
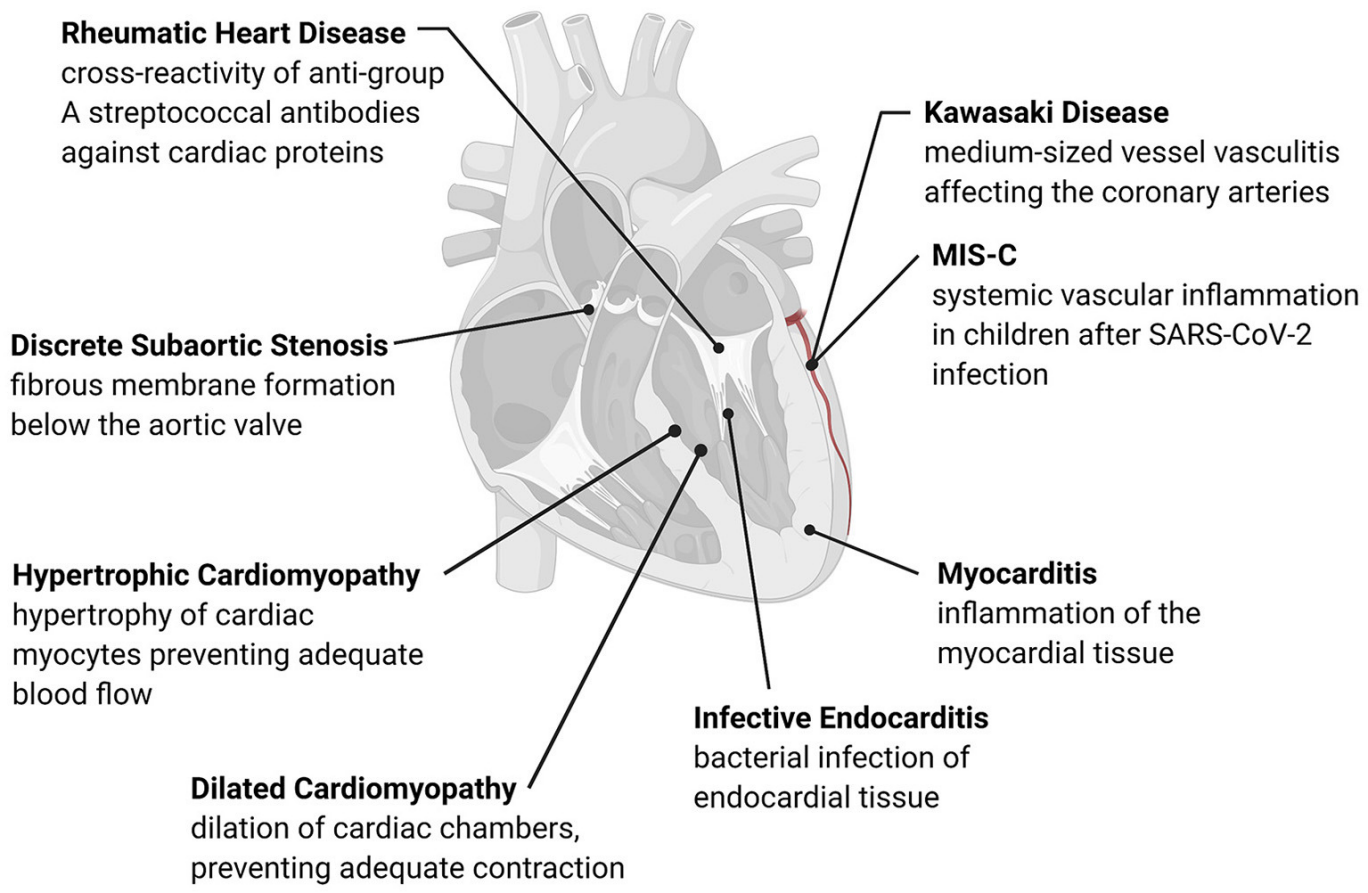
Overview of acquired pediatric heart disease. Acquired pediatric heart diseases can arise as a reaction to various stresses, including flow differences, structural changes, or pathogens. The development of many of these conditions has an immune or inflammatory trigger that causes remodeling of the cardiac structures or activation of native immune cells. Many of these conditions are progressive, leading to long-term complications such as valve damage, fibrosis, and adverse cardiac remodeling.

Building upon information from adult cardiac disease and the associated immune and inflammatory responses, we can further develop our understanding of pediatric disease. In this review, we will focus on the acquired pediatric cardiac diseases depicted in Figure 1 that have an established immune or inflammatory component in their development or progression. Due to the significant overlap between immune and inflammatory mechanisms in each condition, we have organized the diseases discussed by similarities in their pathogenesis. The following sections include an overview of subsets of acquired pediatric heart disease separated according to current knowledge of their underlying immune mechanisms, including pathogen-induced structural cardiac disease, myocardial inflammatory disease, inflammation-mediated cardiac fibrosis, and inflammation-mediated vascular disease, summarized in Table 1. Within each category, we highlight the role of both the immune and inflammatory responses in disease development. This understanding of disease pathophysiology can inform mechanisms underlying the development of pediatric heart disease, its progression, and best diagnostic and therapeutic practices.

**Table 1:** Immune and Inflammatory markers associated with acquired heart disease.

**Disorder**	**Mechanisms**	**References**
Rheumatic heart disease	- ↑ Presence of T lymphocytes within rheumatic valve tissue - ↑ Circulating T lymphocytes - ↑ VCAM-1 expression - ↑ Acute phase reactants (CRP, Homocysteine) - ↑ Cytokine (IL-6, TNF-α) production	Raizada et al. (11), Guilherme et al. (12), Habeeb and Al Hadidi (13), Toor and Vohra (14), Wen et al. (15), Sarkar et al. (16), Rastogi et al. (17), Sikder et al. (18)
Endocarditis	- ↑ Immune complexes (complement protein, antibodies)	Boils et al. (19)
Myocarditis	- ↑ Macrophage activation - ↑ IFN-γ release by NK cells - ↑ Leukocyte counts	Morimoto et al. (20), Caughey et al. (21), Ong et al. (22)
Dilated cardiomyopathy	- ↑ Circulating autoantibodies - ↑ Cytokine (IFN-γ, TNF-α) production	Muir et al. (23), Felix et al. (24), Muller et al. (25), Caforio et al. (26), Jin et al. (27), Balci et al. (28)
Hypertrophic cardiomyopathy	- ↑ NF-κB - ↑ Acute phase reactants (CRP) - ↑ Cytokine (IL-1, IL-6, TNF-α) production - ↑ Galectin-3 - ↑ ROS - ↑ MCP-1	Högye et al. (29), Zen et al. (30), Dimitrow et al. (31), Kuusisto et al. (32), Fang et al. (33), Emet et al. (34)
Discrete subaortic stenosis	- ↑ Macrophage and monocyte activation - ↑ NF-κB - ↑ AP-1 - ↑ ROS	Chistiakov et al. (35), Masse et al. (36)
Kawasaki disease	- ↑ Lymphocytes - ↑ IgA plasma cells - ↑ Neutrophils - ↑ Acute phase reactants (CRP) - ↑ Cytokine (IL-6, IL-10, IFN-γ) production	Burns et al. (37), Anderson et al. (38), Brown et al. (39), Agarwal and Agrawal (40), McCrindle et al. (41)
Multisystem inflammatory syndrome in children	- ↑ Acute phase reactants (CRP) - ↑ D-Dimer - ↑ Cytokine (IL-6) production	Belhadjer et al. (42)

*↑ means increased levels / activity*.

## Pathogen-Induced Acquired Heart Disease

Many acquired pediatric cardiac diseases are caused by known pathogens. In the case of rheumatic heart disease (RHD) and endocarditis, cardiac valve damage results from either direct cardiac infection, as seen in infective endocarditis, or complications caused by an immune response to bacterial pathogens, as seen in RHD (43–46). Defining the immune and inflammatory responses at play in these conditions can improve screening and diagnostic techniques and mitigate long-term valve damage to reduce the global health burden of pediatric heart disease.

### Rheumatic Heart Disease

**Figure 2:**
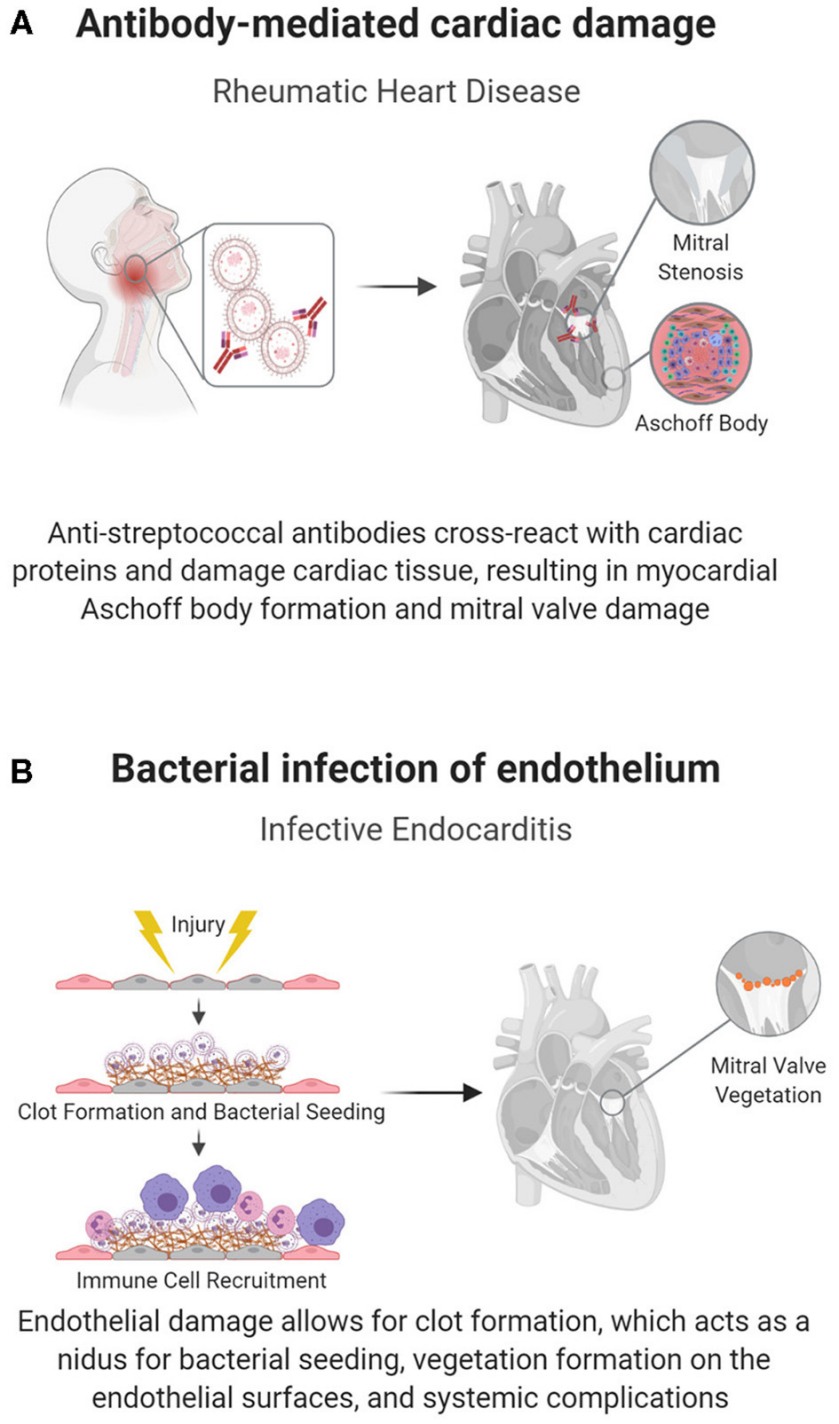
Pathogen-induced acquired pediatric heart disease. Some pediatric cardiac conditions have well-defined immune causes in response to an infectious organism. **(A)** In RHD, biomimicry, in which antibodies against Group A streptococcal bacteria cross react with cardiac proteins, causes cardiac damage, specifically mitral valve fibrosis and subsequent stenosis. **(B)** The endothelium can also become directly infected, as seen in endocarditis, in which flow induced damage creates a nidus for bacterial seeding.

Antibody-mediated rheumatic heart disease (RHD) is one of the main causes of pediatric heart disease globally, accounting for over 200,000 new diagnoses and deaths each year (47). Unlike most pediatric cardiac diseases, the mechanisms underlying RHD and preventative treatments are relatively well-understood. RHD develops as a complication of inadequately treated Group A streptococcal (GAS) pharyngitis, where antibodies against streptococcal M protein cross-react with cardiac α-helical proteins in a process known as biomimicry (43), shown in Figure 2A. This reaction induces valvular fibrosis and calcification, leading to a characteristic “fish mouth” appearance of the mitral valve and clinical mitral valve stenosis (44).

Although the treatment of GAS pharyngitis with penicillin can prevent its progression into RHD, it is still a major cause of childhood death worldwide due to challenges in health care infrastructure and antibiotic availability (48). Understanding the role of the immune response in disease progression can therefore be beneficial as a target of future research in the diagnosis or treatment of RHD.

Recent studies have shown that in addition to biomimicry, cellular immune responses play a role in the development of RHD (49). These immune responses can cause carditis, but most significantly lead to mitral valve disease (50) as both antibodies and T-cells target cardiac myosin, valvular endothelial cells, the basement membrane, laminin, and vimentin (51). Damaged valve tissue in RHD is characterized by the abnormal presence of T-lymphocytes and the infiltration of helper CD4^+^ and cytotoxic CD8^+^ T-cells. The involvement of the immune system is further supported by the pathognomonic histologic finding of myocardial Aschoff bodies, granulomatous lesions that contain specific monocytes known as Anitschkow cells, and other immune cells (44). Patients with RHD also have circulating cross-reactive T-lymphocytes targeting both streptococcal antigens and cardiac tissue (12).

Beyond the initial antibody or lymphocyte insult to the valve, inflammation can promote disease progression. Damage to endothelial cells increases Vascular Cell Adhesion Molecule (VCAM) expression, allowing for improved T-cell adherence and localized, progressive inflammation (18). This inflammatory response involves the expression of proinflammatory molecules including Interleukin (IL)-6, Tumor Necrosis Factor (TNF)-α, IL-8, IL-2, and acute phase reactants (52); additionally, it is chronically supported by higher levels of the inflammatory markers C-reactive protein (CRP) and homocysteine (13). Peripheral levels of both IL-6 and TNF-α are positively correlated with severe valvular disease and calcification (53). Though larger scale studies of these and other markers are necessary to generalize the role of cytokines throughout the course of disease, they may be promising as predictors of the extent of valve damage, and thereby be beneficial to the early diagnosis and monitoring of RHD progression.

### Infective Endocarditis

Infective endocarditis, or infection of the endocardial layer of the heart, is rare in children and mainly affects children with CHDs with prior surgical repairs, indwelling cardiac devices, or central venous catheters (45, 46). When it manifests, it can have devastating consequences due to bacterial biofilm formation, valvular damage, and potential infectious emboli and bacteremia, all of which necessitate prolonged antibiotic regimens (54).

The causative bacteria, typically gram-positive cocci (Staphylococcus aureus, viridans group streptococcus, enterococcus), have specialized adhesin proteins that facilitate adherence to the cardiac endothelium; however, adhesion is often dependent on endothelial damage, as seen in the abnormal flow patterns of CHD (45, 46). Damage to the endothelium exposes thromboplastin and tissue factor, inducing fibrin deposition, and the development of a non-bacterial thrombotic endocarditis (NBTE). Bacteria can then attach at the NBTE and replicate, initiating infective endocarditis (Figure 2B) (54). The infectious nidus can present either on the cardiac wall where abnormal flow jets cause endothelial damage or downstream of the damage, where slow flow patterns cause bacterial stasis, usually on valves in the left heart (45, 54). Structural heart disease also introduces a risk factor for endocarditis, as it is associated with areas of local flow disturbances. Accordingly, children with unrepaired ventricular septal defects (VSDs) have up to a 12% risk of contracting this otherwise rare condition (54). Furthermore, structural disease is often treated with implanted prosthetic devices, which can act as a nidus for bacterial seeding.

Accompanying the immune response to bacterial infection, a major immune complication of infective endocarditis is the development of glomerulonephritis. This outcome is caused by immune complex deposition (including complement proteins and/or antibodies) within the kidney, leading to vascular blockages and renal inflammation (19). Glomerulonephritis presents its own long-term challenges as children face chronic damage to their kidney function. Therefore, reducing the risk of these progressive complications is another area where understanding immune pathways can benefit children with heart disease.

## Inflammation-Mediated Acquired Heart Disease

Myocarditis and dilated cardiomyopathy (DCM) are associated with cardiac inflammation, with DCM involving the remodeling and dilation of cardiac chambers which can result from prolonged myocarditis. Unlike the bacterial causes of RHD and endocarditis, myocarditis and DCM likely have viral triggers that cause myocardial inflammation, and introduce an autoimmune component targeting cardiac tissue (55–57). Understanding the specific immune and inflammatory mechanisms in myocarditis and DCM can lead to targeted treatments and the prevention of chronic complications from these conditions.

### Myocarditis

**Figure 3:**
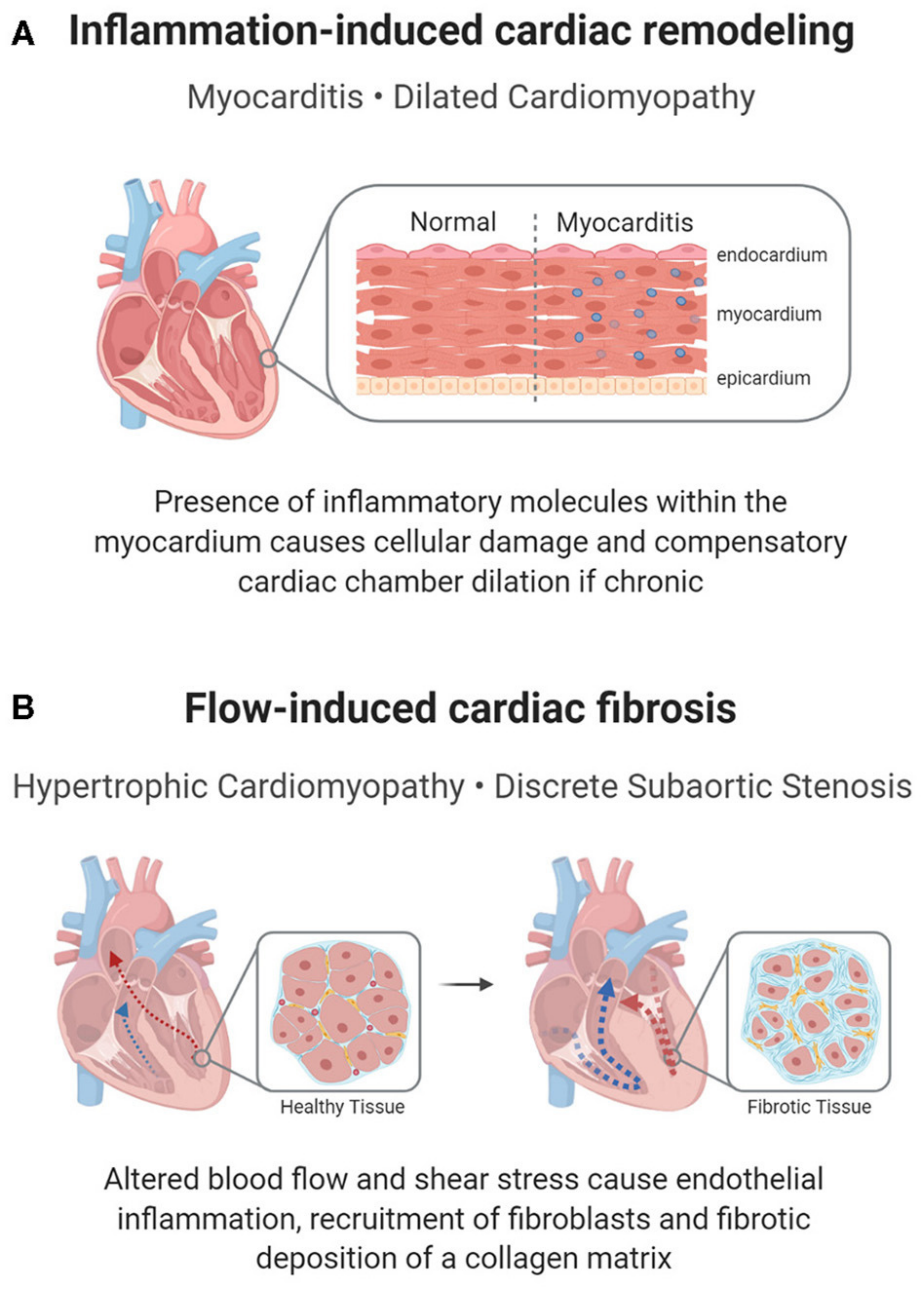
Inflammation-mediated pediatric cardiac disease. In addition to a cellular response, a molecular inflammatory response can lead to pediatric heart disease or its complications. **(A)** In myocarditis, inflammation of the cardiac muscle can cause reactionary remodeling, in some cases leading to DCM. **(B)** Remodeling can also be seen in shear stress induced inflammation, which propagates a pro-fibrotic environment in HCM or DSS.

One form of inflammatory heart disease that presents in a small percentage of children is myocarditis, a disease characterized by the inflammation of the myocardium (Figure 3A). Clinical manifestations vary and most patients experience mild flu-like symptoms or are completely asymptomatic. However, more severe cases may result in acute or chronic heart failure (58), or conduction disturbances such as atrioventricular (AV) blocks (20, 21). Complications from myocarditis are common in children, with up to a 25% mortality rate and only half recovering completely (57).

The pathogenesis of viral and non-viral myocarditis involves infection of the myocardium, immune cell responses, activation of inflammatory pathways, tissue remodeling, and ultimately resolution. Myocarditis can have an infectious or non-infectious etiology. Non-infectious myocarditis, due in part to challenges in identifying infectious agents and data from animal models, is believed to have an autoimmune contribution to disease pathology and progression (56, 59). This is further supported by the association of autoimmune diseases, such as systemic lupus erythematosus (SLE), with myocarditis (56, 60). On the other hand, infectious myocarditis is caused by an identifiable viral agent, often the Coxsackie B virus in children (57). The pathogen triggers an immune response, resulting in myocardial edema and impairment of systolic and diastolic function (58). Infection by viral agents can also trigger macrophage activation and interferon (IFN)-γ release by natural killer (NK) cells, which, when unregulated, result in myocyte injury and cardiac dysfunction (22).

Despite the striking evidence of an immune response, immunosuppressive therapies, such as prednisone and cyclosporine, do not clearly improve the outcomes of myocarditis. In children, the administration of intravenous immunoglobulin (IVIG) has shown promising results in improving outcomes; however, this response has not yet been observed in adults (61, 62). Therefore, more specific anti-inflammatory therapies may be necessary, and the immune mechanisms involved in the propagation of myocarditis should be further investigated for novel treatment options.

Acute episodes of myocarditis can also progress to chronic disease or dilated cardiomyopathy (DCM), discussed below, due to the persistence of pro-inflammatory cytokines that are critical for removing the infectious agent (56). An area for further research is understanding the progression of acute myocarditis into DCM, the frequency of which may be much higher than currently predicted due to limits in viral detection and myocardial biopsy sampling. Furthermore, the histological evidence of myocarditis can deteriorate quickly, introducing challenges in accurately detecting myocarditis as a precursor to DCM. Consequently, only one-third of all cases of pediatric DCM have a known cause, making their subsequent treatment non-specific (58). Understanding this time course and defining key mechanistic targets and therapeutic windows are essential for improving outcomes.

### Dilated Cardiomyopathy

As noted previously, an acute episode of myocarditis can result in DCM. Although cardiomyopathies are generally rare in children, their prognosis is extremely poor, due to limited treatment options and the limited ability to restore native cardiac function. Nearly 40% of children with either symptomatic DCM or hypertrophic cardiomyopathy (HCM) receive a transplant or die within 2 years due to cardiac complications, and survival has improved only slightly over the last two decades (63).

Of those DCM cases with a known etiology, half involve a viral pathogen. Overall, 20% of myocardial biopsies in DCM showed viral infection, with enterovirus and adenovirus being the causative agents (55). Additionally, viral pathogens are frequently credited for the pathogenesis of idiopathic dilated cardiomyopathy (IDCM), and studies have found elevated virus-specific antibody titers in many cases of IDCM (23). Regardless of a viral trigger, most cases of DCM present with cardiac dilation and defective left ventricular or biventricular contraction (64), and are commonly associated with inflammatory responses, including the presence of TNF-α and IFN-γ (28).

Similar to myocarditis, several studies have suggested that autoimmune mechanisms, in addition to a genetic pre-disposition, regulate the pathogenesis of pediatric IDCM. IDCM is hypothesized to be initiated by a pathogenic trigger in patients with pre-disposing human leukocyte antigen (HLA) alleles. Additionally, autoantibody presence in unaffected relatives of patients with IDCM and dysfunctional cardiac activity in 20% of first-degree relatives of DCM patients have been noted. These findings, in addition to associations between HLA-DR4 and both IDCM and anti-cardiac antibodies, support a genetic autoimmune cause (26, 27, 65). Recent studies have also shown that immunosuppression and immunoadsorption reduce the number of circulating autoantibodies and result in improved clinical outcomes, further supporting the relevance of immunological mechanisms in certain cases of DCM development (25). Therefore, investigation into the mechanisms behind the viral presence in DCM is warranted to address potential causative agents and offer prophylactic treatment approaches.

## Inflammation-Mediated Fibrotic Disease

Hypertrophic cardiomyopathy (HCM) and discrete subaortic stenosis (DSS) are both caused by inflammation-mediated remodeling, similar to DCM. In HCM and DSS, localized inflammatory responses are caused by flow disturbances, which lead to the recruitment of inflammatory cells and ultimately, the formation of fibrotic tissue (35, 66, 67). Studying the causes of these conditions can allow for the development of better diagnostics and therapies to prevent chronic remodeling and functional cardiac changes in children.

### Hypertrophic Cardiomyopathy

One of the more severe forms of inflammatory fibrotic remodeling is observed in hypertrophic cardiomyopathy (HCM), a pediatric condition in which septal hypertrophy causes tissue growth into the left ventricle. This growth leads to potential damage to the mitral valve and obstruction of the left ventricular outflow tract (LVOT) (68). HCM places children at risk of acute complications; in fact, HCM is the most common known cause of sudden cardiac death in young athletes, where it accounts for 36% of sudden cardiac deaths resulting from ventricular arrhythmias (5, 69). Many genetic mutations have been identified in children with HCM, typically in genes coding for sarcomere proteins important in cardiac contraction, including *MYH7* and *MYBPC3* (70); however, beyond a genetic pre-disposition, mechanisms of HCM progression have not been clearly defined.

In HCM, the tissue overgrowth includes both hypertrophic cardiomyocytes and fibrotic tissue, indicating that inflammation-induced fibrosis may contribute to hypertrophy (Figure 3B). Histologically, myectomy samples show interstitial and endocardial fibrosis and inflammation, with a disarray of myocytes (71). Clinically, children with HCM have a more pronounced cardiac presence of immune cells and inflammatory molecules (NF-κB, CRP, interleukins, TNF-α), with higher levels corresponding to increased fibrosis (32, 33). The inflammatory response is supported by genetic studies, which show an upregulation of pathways associated with immune cell activation and innate immune cell degranulation in children with HCM (72). Though many cellular processes could account for the inflammatory changes, a proposed mechanism for the inflammatory changes is an increase in neutrophil extracellular traps (NETs) in the left ventricle in children with HCM due to localized flow changes instigating a pro-thrombotic response, which attracts neutrophils (66). Within these NETs, neutrophils release their nuclear contents into the extracellular matrix (ECM) and subsequently trap cells, including inflammatory and fibrotic cells, leading to inflammation, hypoxic and reperfusion injury and fibrosis (73). While localized inflammation, fibrosis, and thrombotic responses caused by NETs have been studied in HCM, further research into the role of NETs in childhood disease pathogenesis could provide a novel approach to defining HCM-associated fibrosis (66).

Markers of endothelial dysfunction, remodeling, and immune cell infiltration are present at even early stages of HCM (74), potentially enabling earlier detection. Galectin-3, a systemic marker of cardiac fibrosis, is elevated in patients with HCM and is significantly higher in patients with a history of cardiac arrest, syncope, fatal arrhythmias, or sudden cardiac death (34). The extent of fibrosis can also be correlated with increases in Stromal Cell-Derived Factor (SDF) and Macrophage Chemoattractant Protein-1 (MCP-1), which increase immune cell recruitment (33), and chronic systemic increases in pro-fibrotic and inflammatory cytokines such as IL-1, TNF-α, and CRP (32).

Inflammatory changes are more prominent in the case of hypertrophic obstructive cardiomyopathy, where myocyte hypertrophy blocks the forward flow of blood. The obstruction-generated flow disruption alters the mechanical stresses experienced by cardiac cells and also leads to changes in the cytokine profile, which subsequently induces myofibroblast differentiation and collagen deposition (75). These cytokine changes include elevations in IL-6 in left ventricular dilation (30) increased MCP-1, and subsequent macrophage recruitment in systolic dysfunction (76), and higher levels of reactive oxygen species (ROS), which disrupt typical endothelial function (31). Since the increased immune and inflammatory presence can act as positive feedback to drive pathologic changes in HCM, the study of molecules along this pathway as therapeutic targets has the potential to prevent adverse cardiac remodeling.

### Discrete Subaortic Stenosis

Similar inflammatory and fibrotic mechanisms have been suggested to cause discrete subaortic stenosis (DSS), a condition in which a fibrotic membrane forms below the aortic valve, obstructing blood flow into the aorta (36, 77). In DSS, an irregular LVOT architecture, such as increased mitral and aortic valve separation or a steep aortoseptal angle, results in abnormal flow patterns and changes in wall shear stress (77). Consequently, this disturbed flow is believed to induce a proliferative fibrotic response from the endothelial cells and resident fibroblasts, resulting in the characteristic membrane formation of DSS (67) (Figure 3B). Histological analysis of septal myectomy in these patients shows endocardial and interstitial fibrosis, evidence of inflammation, vacuolization, and glycosaminoglycan and proteoglycan deposition (78), which implies that the development of this membrane may partly be due to an immune etiology.

Although the mechanism of membrane formation in DSS has not been specifically elucidated, there are hypotheses that indicate that shear stress-induced endothelial damage may cause a fibrotic response, similar to its effects in other tissues. Specifically, it has been shown in vasculature that at areas of elevated flow with high wall shear stress (WSS), the interfacial force caused by blood flow and positive WSS spatial gradients encourage cellular damage and ECM degradation (36, 79). This flow condition then results in the increased endothelial release of microRNA (miR)-155 and miR-205/712 which activate pro-inflammatory phenotypes in macrophages and monocytes, respectively. Additionally, the pro-inflammatory factors NF-κB and AP-1 are upregulated in this state, which increases the expression of adhesion molecules that promote leukocyte attachment to endothelial cells (35).

These inflammatory changes can be mitigated by altering flow characteristics from disturbed to steady flow. Specifically, laminar flow characteristics upregulate the anti-inflammatory factors Krüppel-like factor 2 (KLF2) and nuclear factor erythroid 2-related factor 2 (Nrf2), which can reduce inflammation through the suppression of NF-κB and Activator Protein (AP)-1. Though the inflammatory pathways that respond to endothelial damage have not been studied extensively in pediatric heart disease, studies focused on endothelial responses to changes in wall shear stress suggest a contributory role in the pathogenesis of DSS. Therefore, it is important to better define the interaction between biomechanical forces and fibrosis to understand the fibrotic mechanisms involved, and ultimately prevent the development or progression of these fibrotic conditions.

## Inflammation-Mediated Vascular Disease

Similar to the acquired pediatric cardiac diseases discussed above, pediatric vascular disease also has a likely pathogenic trigger. Both Kawasaki Disease (KD) and multisystem inflammatory syndrome in children (MIS-C) are believed to have a viral cause, leading to inflammatory responses and vascular damage (80, 81). In both cases, the inflammation can have profound implications on the heart, leading to acute conditions, such as heart failure, and long-term complications, including coronary artery aneurysms (81, 82).

### Kawasaki Disease

Kawasaki disease (KD), an acute vasculitis that typically develops in children within the first few years of life as a complication of an infection (80), is the primary cause of acquired pediatric heart disease (83). KD is a clinical diagnosis in children who present with prolonged high fever, rash, extremity swelling, and inflammation of the lips, mouth, and throat. If untreated, it is a critical risk factor for subsequent coronary artery aneurysms and myocardial infarction in early adulthood (58, 84).

The acute inflammatory symptoms of KD suggest an infectious cause, which is further supported by their resolution within 14 days and the young age group in which it presents, namely children under age 5. KD is rarely seen in infants, who are protected by maternal antibodies, and in adults who have likely developed immunity to the causative agent (40). Though no specific pathogenic trigger for KD has been elucidated, there is compelling evidence of immune-mediated damage. For example, the clinical symptoms of a rash and red tongue have been compared to a toxic shock-like reaction, as seen with bacterial superantigen infection. The vascular invasion of cytotoxic T-cells in KD, on the other hand, support a viral etiology (40).

Animal models of KD facilitate research of the immune responses seen in this condition. In murine models, the phenotype of KD is created by exposing mice to extracts from bacteria, such as *Lactobacillus casei* (85, 86), or yeast, such as *Candida albicans* (87). These models provide a promising route to understand the cytokine profiles that lead to vascular damage, such as increased IL-1 and granulocyte macrophage colony stimulating factor (GM-CSF) (86, 88), and possible therapeutic options (85, 87).

**Figure 4:**
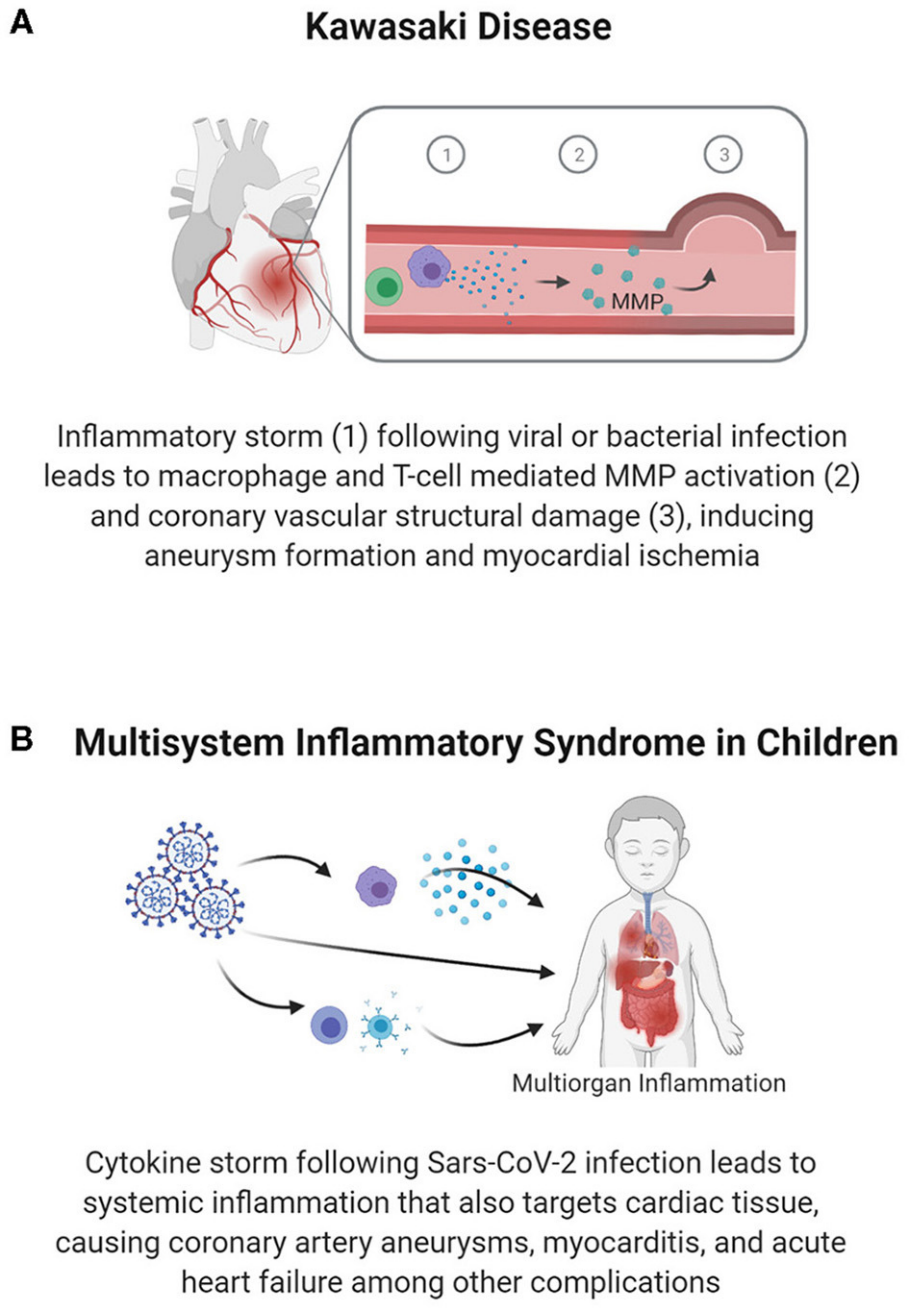
Vascular inflammation-mediated pediatric cardiovascular disease. Inflammation following infection can lead to vascular damage and cardiac pathology. Inflammation and an associated immune response within the vasculature can cause vascular structural damage, as seen in coronary aneurysms in Kawasaki disease **(A)**, which reduces blood flow in the coronary arteries and predisposes individuals to premature MI. In Multisystem Inflammatory Syndrome in Children **(B)**, systemic inflammation causes damage to various organ systems, including targeting vascular and cardiac tissues to cause myocarditis, coronary artery aneurysms, and heart failure.

Through clinical studies and studies in animal models, researchers have found that the complications of KD have substantial immune and inflammatory components. Along with the clinical presentation of fever, rash, and conjunctivitis, there is a markedly elevated expression of markers of systemic inflammation (i.e., CRP and IFN-γ) and the upregulation of inflammatory cytokine (IL-6, IL-10) production (40, 41). Vascular damage, which is a critical consequence of KD, may involve increased T-cell activity, increased effector T-cell specific cytokines, and reduced regulatory T-cell responses (40). In cases of coronary damage, additional immune cells, such as neutrophils, lymphocytes (specifically cytotoxic CD8^+^ T-cells), and IgA plasma cells are seen at the coronary lesion sites (39). These T-cells and macrophages within the coronary arteries secrete TNF-α, which activates matrix metalloprotease (MMP)-9 to degrade extracellular matrix components, reducing the integrity of vasculature and pre-disposing children to aneurysm formation (40) (Figure 4A). Therefore, understanding and treating the long-term vascular inflammatory profile could prevent the development of coronary complications and potential cardiac ischemia resulting from KD.

The role of the immune system is further supported by the use of IVIG as an effective form of treatment (58). It is hypothesized that IVIG may target pathogenic antigens, regulate cytokine production, and/or suppress effector T-cell activity and antibody production (40). Better elucidating these mechanisms along with the cause of KD can allow for potential advancements in disease intervention, especially for KD that is refractory to IVIG treatment or KD that is not treated acutely.

### Multisystem Inflammatory Syndrome in Children

In the midst of the COVID-19 pandemic, a sudden spike in cases of a Kawasaki-like disease known as multisystem inflammatory syndrome in children (MIS-C) has been reported (89, 90). The similarities of MIS-C to KD and the pathways causing MIS-C are still being studied. Nonetheless, the role of a robust inflammatory response has been identified. As shown in Figure 4B, current data indicates multi-organ inflammatory involvement in MIS-C, with children suffering from gastrointestinal, respiratory, hematologic, neurologic, and cardiac symptoms presenting approximately 4 weeks after infection with SARS-CoV-2 (81, 91). Relative to KD, patients with MIS-C are more likely to present with myocardial complications, including ventricular dysfunction, arrhythmia, and shock (81). The collective set of symptoms leads to 75% of children with MIS-C being admitted to the PICU, and 4–5% requiring EMCO (82).

The cardiac complications of MIS-C vary, but range from pericarditis to vascular damage, including coronary artery aneurysms, and heart failure (81). The more severe end of the spectrum includes children who present with febrile cardiogenic shock or left ventricular dysfunction in conjunction with systemic inflammation and endothelial dysfunction (42), as evidenced by the elevated inflammatory markers CRP and D-Dimer. The immune and inflammatory responses are characterized by an IL-6 and IFN-γ cytokine storm, macrophage activation, elevated neutrophil counts, lymphopenia, and an elevated neutrophil: lymphocyte ratio (92, 93). Additionally, >70% of children with MIS-C show increased biomarkers of cardiac damage or dysfunction, such as natriuretic peptides and/or cardiac troponin (82). Recent studies have also correlated the need for intensive care in children with MIS-C and the levels of systemic inflammatory markers (94); though these are correlations based on current clinical data, these inflammatory markers may have utility in predicting the severity of MIS-C associated cardiac complications in children as further studies are conducted.

Similar to KD, treatment for the majority of patients with MIS-C consists primarily of IVIG administration, whereas patients with persistent inflammatory states are treated with an IL-1 receptor antagonist (42). There is a greater resistance to IVIG in MIS-C relative to KD (82), necessitating improved therapeutics. Therefore, in the face of this novel immune complication, a better understanding of the underlying immune mechanisms can help predict long-term cardiac complications and appropriate treatment regimens.

## Conclusion

Despite their prevalence and high clinical impact, the mechanisms causing many pediatric heart diseases have yet to be elucidated. This knowledge gap may be due to the heterogeneity of the causes of these diseases and the broad spectrum of phenotypes. Despite the limited understanding of these conditions, there is substantial support for the role of immune and inflammatory responses in causing these heart diseases or their complications. The major mechanism linking the two is an increase in pro-inflammatory mediators and immune cell recruitment, which can foster an autoimmune response against cardiac proteins, induce cardiac remodeling, cause fibrosis due to changes in flow patterns, and/or stimulate vascular inflammatory responses. However, complicating our basic understanding of these disease processes is the complexity of the immune system and the diverse outcomes that can result from an immune response, making the determination of causative immune pathways difficult.

Applying immune mechanisms from adult cardiac disease can provide the foundation to studying immune and inflammatory mechanisms in children. For example, cardiac remodeling after a myocardial infarction (MI) in adults relies on a delicate balance of pro- and anti-inflammatory markers and the temporal control of immune cell recruitment, such as macrophages, neutrophils, and T-lymphocytes (7, 9, 95). Similar mechanisms can be explored in pediatric conditions involving cardiac remodeling or fibrotic healing responses. Moreover, adult cardiac disease can inform us on research methods applicable to pediatric diseases. For example, the development of murine models of MI has allowed for *in vivo* studies of immune responses in cardiac tissue (96). Additionally, immune specific modeling, such as multi-scale modeling of leukocyte transendothelial migration (TEM) during atherogenesis combine spatiotemporal events that occur at the cellular level to identify the role of leukocytes in atherosclerotic plaque evolution (97).These techniques not only provide the potential to extrapolate findings from adults to better model pediatric disease, but also prevent the development of adult-onset complications, including MI or peripheral vascular disease.

Furthermore, as pediatric heart diseases become better understood, and the role of the immune system and inflammation clarified, there may be a potential to treat pediatric heart disease with immune-modulating therapies. For example, targeted anti-inflammatories that mitigate the immune response have shown benefits in reducing adverse cardiac remodeling in adults (98, 99). Some of these therapies may also be applicable in children and can help reduce the long-term risks of pediatric heart disease; however, the use of current and future immunomodulatory therapies in the children relies on a more holistic understanding of pediatric heart disease. Therefore, it is imperative that we clarify the precise contribution of the immune response in individual cardiac conditions to improve the quality-of-life and reduce the risk of fatal cardiac complications in children with heart disease.

## Author Contributions

KG-A conceptualized the article. EJ and KLS conducted the research and drafted the manuscript. All authors critically edited the article for intellectual merit.

## Conflict of Interest

The authors declare that the research was conducted in the absence of any commercial or financial relationships that could be construed as a potential conflict of interest. The reviewer EA declared a shared affiliation, with no collaboration, with the authors KLS and YN to the handling editor at the time of the review.

## Publisher's Note

All claims expressed in this article are solely those of the authors and do not necessarily represent those of their affiliated organizations, or those of the publisher, the editors and the reviewers. Any product that may be evaluated in this article, or claim that may be made by its manufacturer, is not guaranteed or endorsed by the publisher.

## Abbreviations

CRP: C-reactive protein
ECM: extracellular matrix
IFN: interferon
IL: interleukin
IVIG: intravenous immunoglobulin
MCP: macrophage chemoattractant protein
MMP: matrix metalloprotease
NF-κB: Nuclear Factor–κB
ROS: reactive oxygen species
TNF: tumor necrosis factor.
